# An observational study of breakthrough SARS-CoV-2 Delta variant infections among vaccinated healthcare workers in Vietnam

**DOI:** 10.1016/j.eclinm.2021.101143

**Published:** 2021-09-30

**Authors:** Nguyen Van Vinh Chau, Nghiem My Ngoc, Lam Anh Nguyet, Vo Minh Quang, Nguyen Thi Han Ny, Dao Bach Khoa, Nguyen Thanh Phong, Le Mau Toan, Nguyen Thi Thu Hong, Nguyen Thi Kim Tuyen, Voong Vinh Phat, Le Nguyen Truc Nhu, Nguyen Huynh Thanh Truc, Bui Thi Ton That, Huynh Phuong Thao, Tran Nguyen Phuong Thao, Vo Trong Vuong, Tran Thi Thanh Tam, Ngo Tan Tai, Ho The Bao, Huynh Thi Kim Nhung, Nguyen Thi Ngoc Minh, Nguyen Thi My Tien, Nguy Cam Huy, Marc Choisy, Dinh Nguyen Huy Man, Dinh Thi Bich Ty, Nguyen To Anh, Le Thi Tam Uyen, Tran Nguyen Hoang Tu, Lam Minh Yen, Nguyen Thanh Dung, Le Manh Hung, Nguyen Thanh Truong, Tran Tan Thanh, Guy Thwaites, Le Van Tan

**Affiliations:** 1 Hospital for Tropical Diseaes, Ho Chi Minh City, Vietnam; 2 Oxford University Clinical Research Unit, Ho Chi Minh City, Vietnam; 3 Centre for Tropical Medicine and Global Health, Nuffield Department of Medicine, University of Oxford, Oxford, UK

**Keywords:** Delta variant, Oxford-AstraZeneca, COVID-19, vaccine breakthrough, Vietnam

## Abstract

**Background:**

Data on breakthrough SARS-CoV-2 Delta variant infections in vaccinated individuals are limited.

**Methods:**

We studied breakthrough infections among Oxford-AstraZeneca vaccinated healthcare workers in an infectious diseases hospital in Vietnam. We collected demographic and clinical data alongside serial PCR testing, measurement of SARS-CoV-2 antibodies, and viral whole-genome sequencing.

**Findings:**

Between 11^th^–25^th^ June 2021 (7-8 weeks after the second dose), 69 staff tested positive for SARS-CoV-2. 62 participated in the study. Most were asymptomatic or mildly symptomatic and all recovered. Twenty-two complete-genome sequences were obtained; all were Delta variant and were phylogenetically distinct from contemporary viruses obtained from the community or from hospital patients admitted prior to the outbreak. Viral loads inferred from Ct values were 251 times higher than in cases infected with the original strain in March/April 2020. Median time from diagnosis to negative PCR was 21 days (range 8–33). Neutralizing antibodies (expressed as percentage of inhibition) measured after the second vaccine dose, or at diagnosis, were lower in cases than in uninfected, fully vaccinated controls (median (IQR): 69.4 (50.7-89.1) vs. 91.3 (79.6-94.9), p=0.005 and 59.4 (32.5-73.1) vs. 91.1 (77.3-94.2), p=0.002). There was no correlation between vaccine-induced neutralizing antibody levels and peak viral loads or the development of symptoms.

**Interpretation:**

Breakthrough Delta variant infections following Oxford-AstraZeneca vaccination may cause asymptomatic or mild disease, but are associated with high viral loads, prolonged PCR positivity and low levels of vaccine-induced neutralizing antibodies. Epidemiological and sequence data suggested ongoing transmission had occurred between fully vaccinated individuals.

**Funding:**

Wellcome and NIH/NIAID

**Research in context**

**Evidence before this study**

To date, existing data showed that breakthrough Delta variant infections among vaccinated people had comparable viral loads with those in unvaccinated individuals infected with the Delta variants, but there has been no study comparing viral loads of breakthrough infections with those in cases infected with the original SARS-CoV-2 strains detected in early 2020. Using PCR or viral culture, previous reports also showed that cases of breakthrough infections had a short duration of viral shedding of 7 days or less, but none has reported robust evidence demonstrating the transmission of SARS-CoV-2 between fully vaccinated people. Most recently, a study in Israel identified a correlation between neutralizing antibody titers after the second dose and at diagnosis and breakthrough infection with the Alpha variant.

**Added value of this study**

This study examined 62 (asymptomatic (n=13) and mildly symptomatic (n=49)) cases of breakthrough Delta variant infections among Oxford-AstraZeneca vaccinated healthcare workers in an infectious diseases hospital in Ho Chi Minh City, Vietnam, and demonstrated evidence of secondary transmission between vaccinated individuals through the analysis of epidemiological and viral whole-genome sequence data. Peak viral loads assessed by Ct value were 251 times higher than those in cases infected with the original SARS-CoV-2 strain detected in Vietnam between March and April 2020. Vaccine-induced neutralizing antibodies after the second dose and at diagnosis were lower than those in the matched uninfected, fully vaccinated controls, but they were not associated with peak viral loads (i.e. infectivity) or the development of symptoms during the course of infection.

**Implications of all the available evidence**

Breakthrough Delta variant infections are associated with asymptomatic or mild disease, but resulted in high viral loads and prolonged PCR positivity. In this study, high viral loads coupled with a poorly ventilated indoor setting without in-office mask wearing might have facilitated the transmission of the Delta variant between vaccinated individuals, emphasizing that social distancing measures remain critical to reduce the transmission of SARS-CoV-2 Delta variant, event in countries where vaccination coverage is high. The absence of correlation between neutralizing antibody levels and peak viral loads suggested that vaccine might not lower the transmission potential of breakthrough infection cases.

Alt-text: Unlabelled box

## 1 Introduction

SARS-CoV-2 Delta variant is approximately 60% more transmissible than the Alpha (B.1.1.7) variant, and has rapidly spread worldwide [1], posing a significant threat to global COVID-19 control. The Delta variant possesses mutations in the spike protein (including L452R and T478K) that makes the virus less susceptible to neutralizing antibodies generated by current vaccines or natural infection [2,3]. These features have raised concern about Delta variant vaccine escape potential and breakthrough infections.

Data on vaccine breakthrough infections, especially those caused by the Delta variant, are limited [4,5]. Likewise, little is known about the associated serological markers (i.e. vaccine induced neutralizing antibody levels) of breakthrough infections, especially those infected with the Delta variant [6]. And scarce epidemiological and molecular data exist regarding to the transmission of SARS-CoV-2 delta variant between fully vaccinated individuals. Such knowledge is critical to informing the development and deployment of COVID-19 vaccine, and the implementation of infection control measures.

Between 11^th^ and 25^th^ June 2021, an outbreak of breakthrough infections occurred among staff members of an infectious diseases hospital, the Hospital for Tropical Diseases (HTD), in Ho Chi Minh City, Vietnam. Nearly all of the staff had received a second dose of Oxford-AstraZeneca vaccine 7 weeks previously. The first case (patient 1), a 41-year old man, was identified by PCR on 11^th^ June 2021 having experienced body pain and tiredness. Following the diagnosis of patient 1, HTD expanded the PCR screening for SARS-CoV-2 to all staff members. By the end of 12^th^ June 2021, 52 additional members were found to be infected, including all 6 members who shared an office with patient 1.

Following Vietnamese Government regulations, HTD was locked down for two weeks (12^th^-26^th^ June 2021), with no one allowed to enter or leave the hospital. Further PCR testing of all staff during this period identified 16 additional positive cases, totaling 69 infected members from 20/34 departments, corresponding to an overall PCR positive rate of 8% (69/866) at the hospital-wide level. Serological testing for SARS-CoV-2 N protein antibodies (indicating naturally acquired infection) was carried out on 683 staff (including those who stayed in HTD during the lockdown and the infected cases) between 13^th^ and 16^th^ June 2021, but none was positive.

Here, we report the results of in-depth investigations of the outbreak. We focused our analysis on the clinical features, viral evolution and dynamics of viral loads and antibody responses during the course of breakthrough infections.

## 2 Materials and methods

### 2.1 Setting

HTD is a 550-bed tertiary referral hospital for patients with infectious diseases in southern Vietnam [7]. HTD has around 900 members of staff and 34 departments. All offices except one are equipped with air conditioners that recirculate the air without mechanical ventilation (Supplementary Figure 1).

As an infectious hospital, HTD is responsible for COVID-19 patient management. At the time of the outbreak, three departments were dedicated for COVID-19 patients, and around 70 patients were being treated at HTD. Access to COVID-19 patient departments was restricted to medical staff providing COVID-19 patient care. During the lockdown, HTD temporarily stopped receiving new patients, including those with COVID-19.

### 2.2 The vaccine evaluation study: a brief description

**Figure 1:**
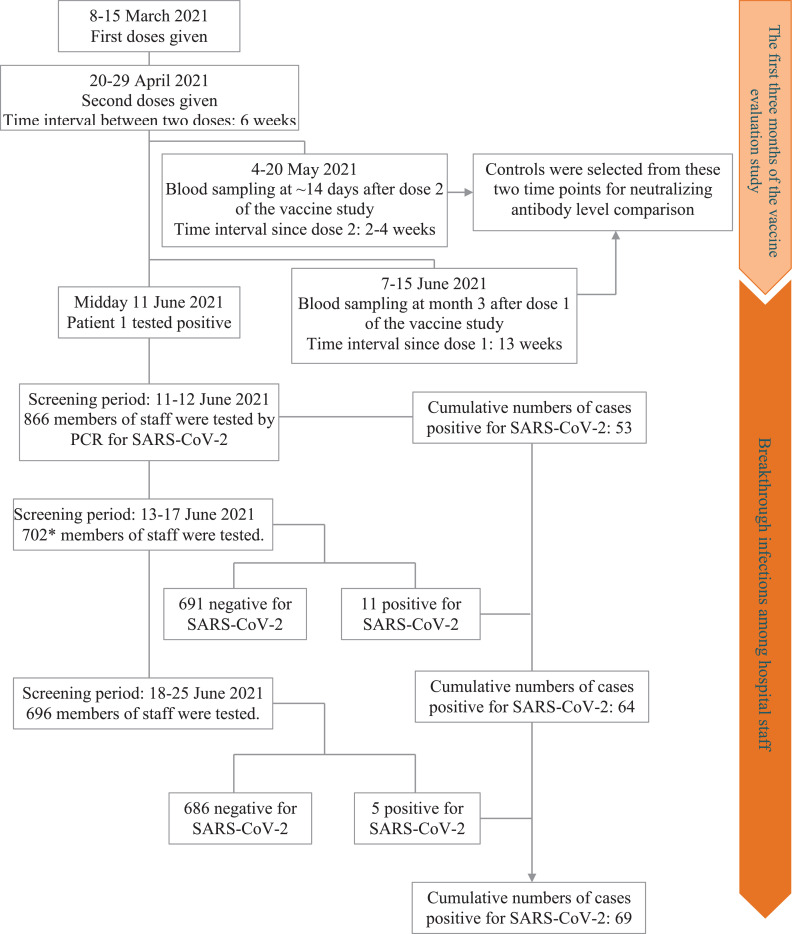
Flowchart showing timelines and results of SARS-CoV-2 RT-PCR screening before and during the lockdown (11-25 June 2021). **Notes to**Figure 1: *The remaining members of staff were working from home.

HTD staff members were amongst the first people in Vietnam to be offered the Oxford-AstraZeneca COVID-19 vaccine, and 554 vaccinated staff have been participating in an ongoing vaccine evaluation study since March 2021. The detailed descriptions about the study was previously reported [8]. In brief, the first vaccine doses were given on 8^th^ March 2021; the second doses were given in the last two weeks of April 2021. The window time between the two doses was 6 weeks (Figure 1). Blood sampling was scheduled for the time points before and approximately at 14 days after each dose, and at week 4, and month 3, 6, and 12 after the first dose. The outbreak coincided with the blood-sampling schedule at month 3 after dose 1 (i.e. between 8^th^–15^th^ June 2021, Figure 1).

### 2.3 The breakthrough infection study

#### 2.3.1 Data and sample collection

We collected demographic, vaccination history, and clinical data alongside the results of routine SARS-CoV-2 PCR testing from the study participants, conducted at least once every three days during hospitalization.

#### 2.3.2 Nasopharyngeal-throat swab collection, PCR testing and viral load conversion

Nasopharyngeal swabs were collected and placed in 1mL of viral transport medium, and 200uL was used for viral RNA extraction using the MagNApure 96 platform (Roche Diagnostics, Germany), according to the manufacturer's instructions. For SARS-CoV-2 RNA detection, we used real-time RT-PCR assay with primers and probe targeted at the envelope protein-coding gene (TIB MOLBIOL) [9] with a cutoff of 40 cycles. PCR Ct values were converted to RNA loads using an in-house established formula (y = -0.3092x + 12.553, R² = 0.9963, where y is viral load and x is Ct value) based on 10-fold dilution series of in-vitro transcribed RNA [9,10].

#### 2.3.3 Whole genome sequencing and sequence analysis

Whole-genome sequences of SARS-CoV-2 were directly obtained from leftover RNA after PCR testing using ARTIC protocol [11] and Illumina reagents on a MiSeq platform with the inclusion of a negative control in every sequencing run. The obtained reads from individual samples were mapped to a SARS-CoV-2 reference genome (GISIAD sequence ID: EPI_ISL_1942165) to generate the consensuses using Geneious software (Biomatter, New Zealand). SARS-CoV-2 variant assignment was carried out using Pangolin [12]. Detection of amino acid changes as compared to the original Wuhan strain was done using COV-GLUE [13]. Maximum likelihood phylogenetic tree was reconstructed using IQ-TREE [14].

#### 2.3.4 Blood collection and SARS-CoV-2 antibody measurement

For SARS-CoV-2 antibody measurement, we obtained 2ml of EDTA plasma from each study participant at diagnosis and every week after diagnosis for a duration of up to three weeks. Any study participants, who were discharged before the time point scheduled for weekly sampling, were not subjected to additional blood collection. When convenient, we used leftover blood samples collected as part of the initial investigation of the outbreak.

We measured antibodies (IgM and IgG) against SARS-CoV-2 nucleocapsid (N) protein using Elecsys Anti‑SARS‑CoV‑2 assay (Diagnostics, Germany) with a cutoff index of 1.0, and SARS-CoV-2 neutralizing antibodies using SARS-CoV-2 Surrogate Virus Neutralization Test (sVNT) (GenScript, USA) with a cutoff of 30% [15]. The experiments were carried according to the manufacturers’ instructions.

#### 2.3.5 Additional data for case-control analyses

To assess the potential association between vaccine breakthrough infections and the levels of vaccine-induced neutralizing antibodies, we compared available antibody levels measured after the second dose (approximately 14 days) and at month 3 after the first vaccine dose in fully vaccinated, uninfected staff (controls) with the cases [8]. To ensure the statistical power of the study, we applied a matching ratio of 1:3 (1 case and 3 controls). We matched cases with the uninfected, fully vaccinated controls for age and gender (Supplementary Tables 1).

For viral load comparison, we used previously reported data from SARS-CoV-2 infected cases from the early phase of the pandemic in Vietnam between March and April 2020 (Supplementary Table 2) [7]. Herein, we used the term “original strain” to refer to the SARS-CoV-2 strain detected in those earlier patients.

### 2.4 Data analysis

Data analysis was carried out using SPSS v27 (SPSS Inc, Chicago, US) or Graphpad Prism v9.0.2 (Graphpad Software, California, US). For comparison between groups of categorical data, we used the Fisher exact test for expected frequencies of <5, otherwise, we used the Chi-squared test. The Mann-Whitney U test was used for nonparametric comparisons of data. The repeated mesurement of viral loads and antibodies over the time were tested using ANOVA and the non-parametric Friedman test, respectively. For comparison between the viral loads of the controls and the case patients we used multivariate regression with type-II sum of square and age, gender, symptomatic status and comorbidity as variables in order to control for potential associated confounding effects. For comparison between neutralizing antibodies after vaccination and at diagnosis between cases and control, we applied the same statistical approach with age, gender and the number days since dose two as variables. We also applied the same multivariate regression analysis approach to assess the association between peak viral loads during the course of breakthrough infections and neutralizing antibodies measured at diagnosis.

### 2.5 Ethics

The study was approved by the Institutional Review Board of HTD and the Oxford Tropical Research Ethics Committee, University of Oxford, UK. Written informed consent was obtained from all the participants. Whole-genome sequencing of SARS-CoV-2 formed part of the national response to the COVID-19 outbreak in Vietnam. Accordingly, obtaining informed consent was deemed unnecessary.

### 2.6 Role of the funders

The funders of the study had no role in data collection, analysis, interpretation, writing of the manuscript and the decision to submit the manuscript.

## 3 Results

### 3.1 Demographics and clinical features

In total 69/866 (8%) staff members were found positive for SARS-CoV-2 by PCR testing during the lockdown period. As per the national COVID-19 management policy in Vietnam, all the 69 infected members of HTD staff were admitted to HTD for clinical follow-up. Their associated departments are listed in Supplementary Table 4. They all were invited to participate in an ongoing observational study [7]. And 62 (including 53/53 tested positive on 11^th^-12^th^ June and 9/16 tested positive between 13^th^-25^th^ June) consented to have their clinical features reported. The 7 staff members who did not enter the study were excluded by chance. Comparison between them and those included did not suggest bias was introduced, male/female ratio: 3/4 vs. 33/29, p=0.702 and median age in years (IQR): 36 (27-53) vs. 42 (32-50), p=0.582, respectively.

Of the 62 study participants, two had received one dose of Oxford-AstraZeneca vaccine, and 60 (including patient 1) had received two doses. Two presented with symptoms at diagnosis (11^th^ and 15^th^ June), and 47 developed symptoms between 1-15 days after diagnosis (Supplementary Figure 2). Chest x-ray examination was performed on 34 cases. Of these, three had evidence of pneumonia, including one requiring oxygen supplementation for three days because of shortness of breath. This staff member was fully vaccinated. Otherwise, they all were either mildly symptomatic or asymptomatic (i.e. no observed symptoms during the course of infection) (Table 1). All recovered uneventfully.

**Table 1:** Demographics and clinical characteristics of the study participants

Signs/Symptoms	All cases (n=62)	Male (n=33)	Female (n=29)
Age, y, median (IQR)	41.5 (32-50)	41 (34-50)	43 (29-50)
Occupation, n (%)			
Nurse	13 (21)	5 (15)	8 (28)
Pharmacist	10 (16)	3 (9)	7 (24)
IT	7 (11)	7 (21)	0
Clinician	7 (11)	5 (15)	2 (7)
Accountant	4 (6)	0	4 (14)
Technical staff	3 (5)	3 (9)	0
Cleaner	2 (3)	2 (6)	0
Others^	16 (26)	8 (24)	8 (28)
Symptomatic, n (%)	49 (79)	24 (73)	25 (86)
Acute respiratory infection^^	43 (69)	23 (70)	20 (69)
PCR diagnosis to illness onset, d, (median; IQR)*	4 (2-7)	3 (2-6)	5 (2-8)
Comorbidity#, n (%)	17 (27)	9 (27)	8 (28)
COVID-19 vaccination¥, n (%)	62 (100)	33 (100)	29 (100)
Two doses	60 (97)	33 (100)	27 (93)
One dose	2 (3)	0	2 (7)
Fever, n (%)	17 (27)	9 (27)	8 (27)
Cough, n (%)	23 (37)	19 (58)	14 (48)
Sore throat, n (%)	21 (34)	9 (27)	12 (41)
Runny nose, n (%)	22 (36)	9 (27)	13 (45)
Loss of smell, n %)	24 (39)	14 (42)	10 (35)
Loss of taste, n (%)	5 (8)	3 (9)	2 (7)
Muscle pain, n (%)	17 (27)	13 (39)	4 (14)
Headache, n (%)	12 (19)	6 (18)	6 (21)
Chest pain, n (%)	2 (3)	0	2 (7)
Nausea, n (%)	5 (8)	3 (9)	2 (7
Others, n (%)$	5 (8)	1 (3)	4 (14)
Shortness of breath	2 (4)	0	2 (6)
Pneumonia, n (%)**	3 (5)	0	3 (10)

**Notes to**Table 1:

IQR: interquartile range

^including data entry (n=1), driver (n=4) and specialists with additional details not available (n=11)

^^Runny nose, cough, sore throat, muscle pain, chest pain, chills, or shortness of breath

*Symptomatic cases only

**One requiring oxygen supplementation via nasal cannula for 3 days.

¥ All receiving Oxford-AstraZeneca vaccine; the second doses were given in last 2 weeks of April 2021.

# Overweight (n=6), obese (n=3), hypertension (n=3), hepatitis B (n=2), diabetes (n=1), pregnancy (n=1), diabetes and hepatitis B (n=1).

$ Chills (n=2), sweating (n=1), giddiness (n=1), red eyes (n=1), and diarrhea (n=1)

### 3.2 Viral loads

At diagnosis, median PCR Ct value of the 62 study participants was 31.9 (IQR: 23.3–34.9), equivalent to log_10_ copies per mL of 4.4 (IQR: 3.5–7.1); eleven (21%) of the first 53 cases from 5 different departments had high viral loads (i.e. a Ct value of between 14.0 and 22.6), equivalent to log_10_ copies per mL of 7.3–9.9, including patient 1 and 4/6 members sharing the office with him.

**Figure 2:**
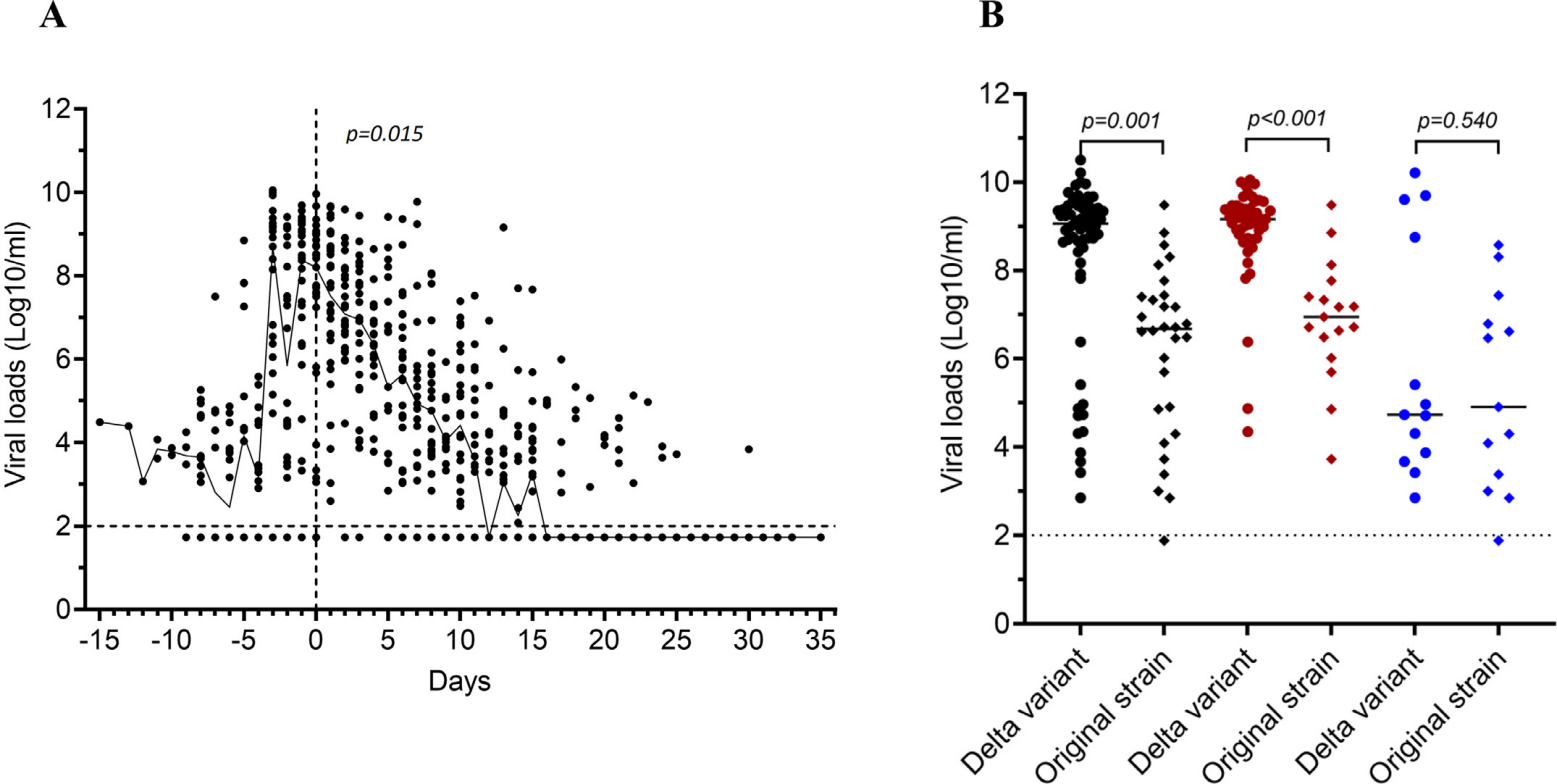
Viral load analyses, A) plot outlining kinetics of viral loads in relation to illness onset of the 49 symptomatic participants, B) comparison between peak viral loads of breakthrough infections (cases) and those (controls) infected with old SARS-CoV-2 strains detected between March and April 2020 in Vietnam. **Notes to**Figure 2: Vertical dashed line indicates the time point of illness onset. Horizontal dashed line indicates detection limit of PCR assay. A) Black lines indicates median viral loads, B) black dots represent for whole groups, red dots represent for symptomatic cases and blue dots represent for asymptomatic cases.

Of the 49 symptomatic cases, high viral loads were observed around 2-3 days before and after symptom onset (Figure 2A). Their peak viral loads measured at any time point during the course of infection were higher than that of asymptomatic cases (median (IQR): 16.5 (15.6–17.9) vs. 30.8 (16.4–33.9), equivalent to median log_10_ viral load of 9.2 copies per mL (IQR: 8.7–9.4) vs. 4.7 copies per mL (IQR: 3.8–9.2), p=0.004, respectively) (Figure 2B and Supplementary Figure 3). The median time from diagnosis to PCR negative was 21 days (range 8–33).

Compared with peak viral loads in SARS-CoV-2 infected cases detected in Vietnam between March and April 2020, median peak viral loads of the breakthrough cases were equivalent to 251 times higher (median log_10_ viral load in copies per mL (IQR): 9.1 (8.7–9.4) vs. 6.7 (4.7–7.4), p=0.001). The differences between the groups were even greater among symptomatic cases (median log_10_ viral load in copies per mL (IQR): 9.2 (8.7–9.4) vs. 7.0 (6.3–7.6), p<0.001). In those without symptoms, however, viral loads were similar (median log_10_ viral load in copies per mL (IQR): 4.7 (3.8–9.2) vs. 4.9 (3.2-8.6) p=0.540) (Figure 2B).

### 3.3 Whole genome sequencing

**Figure 3:**
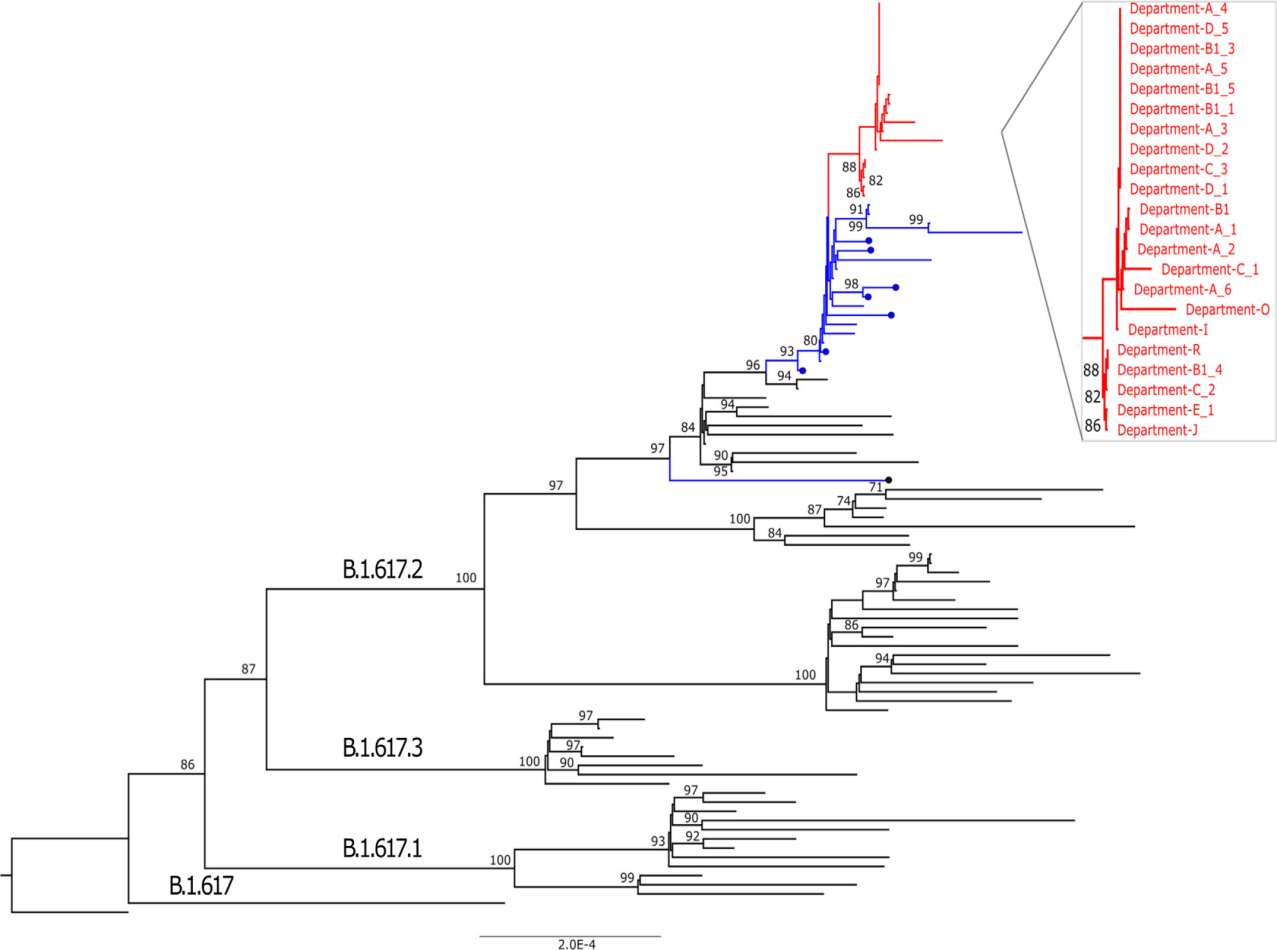
Maximum likelihood tree illustrating the relatedness between SARS-CoV-2 Delta variant strains obtained from cases of vaccine breakthrough infection (red) and contemporary Delta variant sequences obtained from cases of community transmission in Ho Chi Minh City (blue) and from COVID-19 patients admitted to HTD prior to the outbreaks (in blue marked with dots).

A total of 22 whole genome sequences of SARS-CoV-2 were obtained from 22 fully vaccinated staff members (including patient 1 and 1/7 staff member not participating in the present study) from 9 different HTD departments (Supplementary Table 3). All were assigned to the SARS-CoV-2 Delta variant. They were either identical or different from each other by only 1 to 7 nucleotides and no novel amino acid changes were identified among them. Phylogenetically, the 22 sequences clustered tightly together and were separated from the contemporary Delta variant sequences obtained from cases of community transmission in HCMC and from the COVID-19 patients admitted HTD prior to the outbreak (Figure 3).

### 3.4 Antibody development

A total of 210 plasma samples were collected from the 62 study participants, including 61 at diagnosis, and 61 at week 1, 57 at week 2 and 31 at week 3 after diagnosis. The missing data was either attributed to lost to follow up (n=1 each at diagnosis and at week 1 after diagnosis, and n=3 at week 2) or early discharge (n=1 and n=31 at week 2 and 3, respectively).

**Figure 4:**
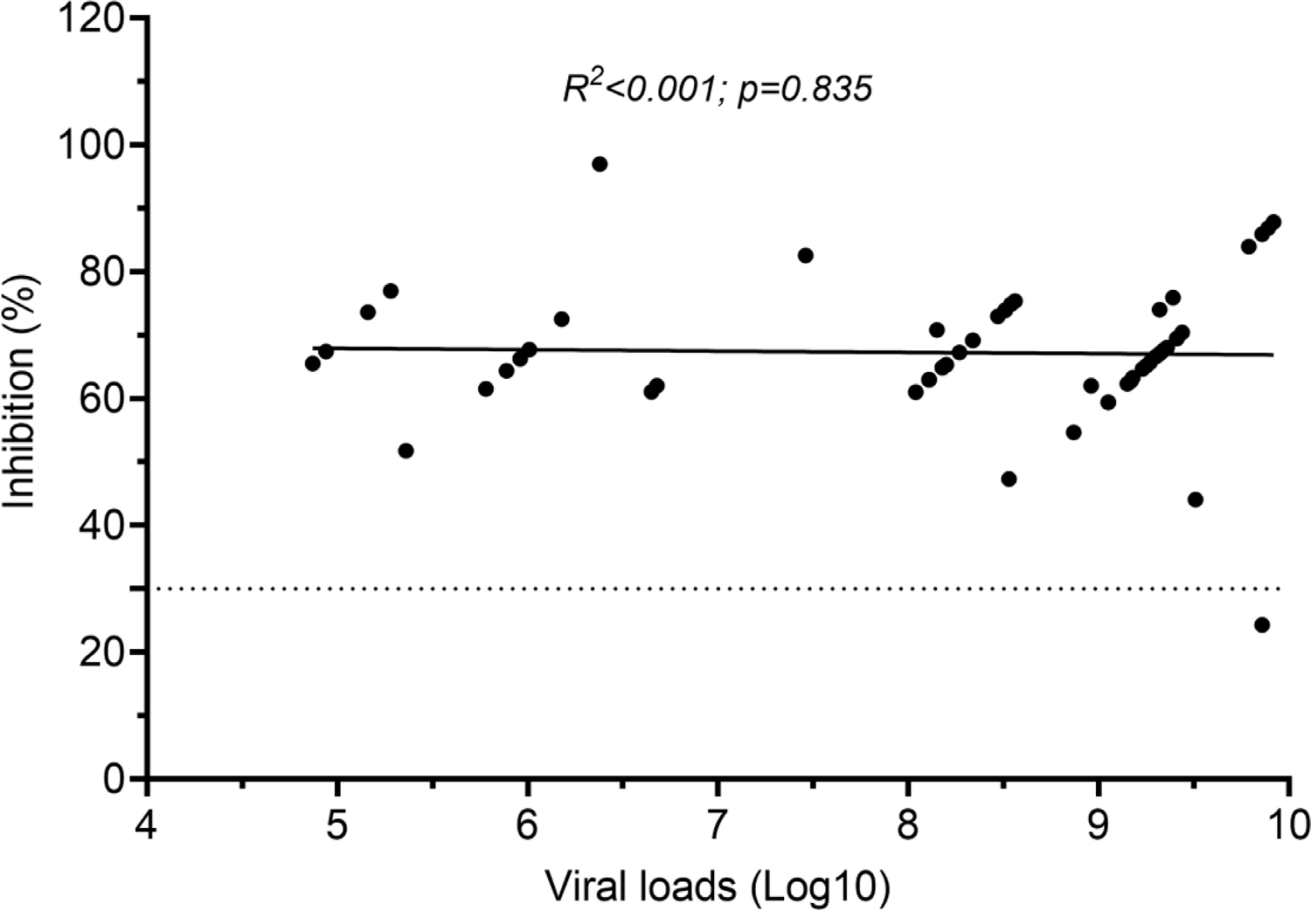
Correlation between neutralizing antibodies at diagnosis and peak viral loads during the course of infection

The 61 at-diagnosis plasma samples were collected 1-3 days before PCR diagnosis (n=7 as part of the initial outbreak investigation between 13^th^ and 16^th^ June 2021), on the day of diagnosis (n=2), and 1 day (n=43) and 2 days (n=9) after diagnosis. Of these, all but three had detectable neutralizing antibodies, with comparable levels between symptomatic and asymptomatic cases (median % of inhibition (IQR): 69.0 (46.5-86.7) vs. 69.7 (61.1-81.8), p=0.918) (Supplementary Figure 4). There was no correlation between neutralizing antibodies at diagnosis and peak viral loads during the course of infection (R^2^<0.001 and p=0.835) (Figure 4). At week 2 and 3 after diagnosis, neutralizing antibody levels of the cases significantly increased (p<0.001, Supplementary Figure 4).

The seroconversion rates for antibodies against N protein steadily increased from 0% at baseline to 65% (20/31) at week 3 after diagnosis. Asymptomatic patients had slightly lower seroconversion rates than symptomatic patients (week 1: 0% (0/13) vs. 4% (2/48), p=1.0, week 2: 0% (0/13) vs. 36% (16/44), p=0.012 and week 3: 40% (2/5) vs. 69% (18/26), p=0.317, respectively) (Supplementary Figure 5).

### 3.5 Case-control analyses of neutralizing antibodies after vaccination and at diagnosis

**Figure 5:**
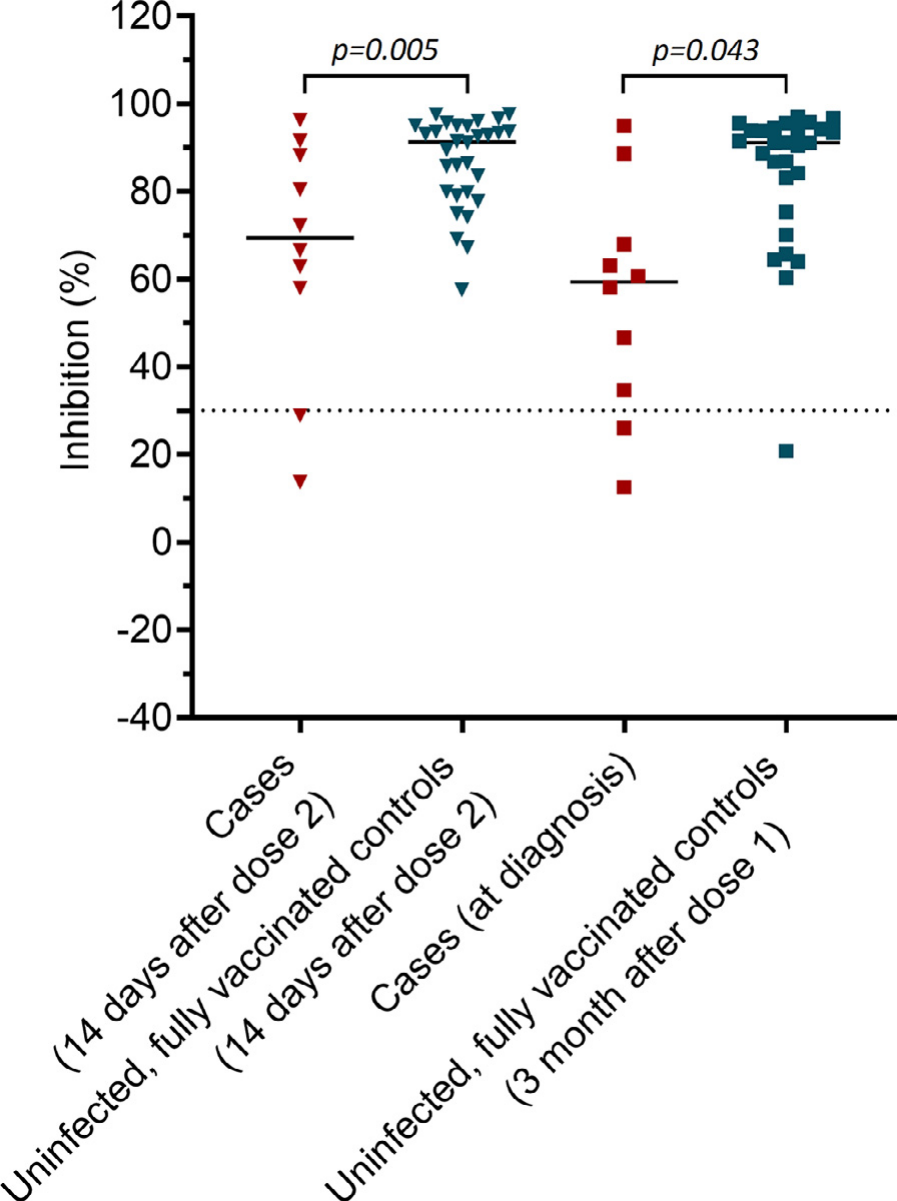
Comparison between neutralizing antibody levels of the 10 case patients (red) and 30 uninfected, fully vaccinated controls (grey green)

Ten of the 62 infected staff members participating in the study had neutralizing antibodies measured at 14 days after their second vaccine dose and at diagnosis (month 3 after the first dose). Neutralizing antibody levels measured at day 14 post vaccination and at diagnosis were similar (median % of inhibition (IQR): 69.4 (50.7-89.1) vs. 59.4 (32.5-73.2), p=0.353). However, compared with the 30 controls (matching ratio 1:3), these 10 cases had lower day-14 and at-diagnosis neutralizing antibody levels (median % of inhibition (IQR): 69.4 (50.7-89.1) vs. 91.3 (79.6-94.9), p=0.005 and 59.4 (32.5-73.1) vs. 91.1 (77.3-94.2), p=0.043, respectively) (Figure 5).

Case-control analysis for all 62 study participants was hampered by the unavailability of the controls. However, there was no difference in demographics and the levels of neutralizing antibodies measured at diagnosis between the 10 infected staff members included for case-control analysis and those who was not included (Supplementary Table 4).

## 4 Discussion

We studied Oxford-AstraZeneca vaccine breakthrough infections associated with SARS-CoV-2 Delta variant among staff in a 550-bed infectious diseases hospital in HCMC, Vietnam between 11^th^ and 25^th^ June 2021. The infections occurred 7-8 weeks after most staff received a second vaccine dose. 62/69 infected staff members participated in the clinical study. One required oxygen supplementation for three days but all other infections were either asymptomatic or mild and everyone made a full recovery, consistent with vaccine's effectiveness at protecting against severe disease [[16], [17], [18], [19]]. Yet, we report for the first time the serological markers associated with breakthrough Delta variant infection, and the likely transmission of the Delta variant between fully vaccinated individuals.

All of the 23 whole-genome sequences of SARS-CoV-2 obtained from the infected, fully vaccinated staff members clustered tightly on a phylogenetic tree and were distinct from contemporary Delta variant genomes obtained from the community and the hospital prior to the outbreak. Furthermore, the outbreak appeared to localize to a poorly ventilated administrative office, where all 7 members were fully vaccinated but became infected. These findings strongly suggest transmission from and between fully vaccinated individuals. We cannot definitively exclude a common external source, however, but other features indicate it was unlikely. At HTD, access to COVID-19 patient departments was restricted to medical staff and the majority of the infections occurred amongst non-medical staff. Additionally, because community transmission of SARS-CoV-2 had been escalating in HCMC since May 2021, public gatherings of more than 10 people had been banned. Thus it seems unlikely that the outbreak originated from hospital patients or was linked to a mass gathering, super-spreader event outside the hospital.

At the beginning of the outbreak (11^th^ June 2021), only patient 1 presented with symptoms. Therefore, secondary transmission may have occurred prior to the development or in the absence of symptoms, a well-recognised phenomenon in unvaccinated people [7]. In our study, transmission may be attributed to several factors. Firstly, high viral loads, >7 log_10_ copies per mL, which has been strongly correlated with positive culture (i.e. infectiousness) [10,20], was recorded at diagnosis in 11 of the first 53 positive cases. Second, HTD offices are typically equipped with air conditioners without mechanical ventilation systems, an indoor setting that could facilitate the transmission of SARS-CoV-2 [5,21]. Third, mask wearing in the office was not mandatory at the time.

At diagnosis neutralizing antibody levels in a subset of the infected staff were comparable with that measured at 14 days after the second vaccine dose. Additionally, prior to the outbreak, HTD staff members remained naïve to the infection, as evidenced by the absence of N protein antibodies in all staff members [22,23]. Therefore, neutralizing antibodies at diagnosis were likely derived from vaccination rather than natural infection.

Lower levels of neutralizing antibodies after vaccination and at diagnosis were associated with breakthrough infections in a recent report from Israel [6], consistent with our findings. However, in contrast with the Israeli study, we found no correlation between vaccine-induced neutralizing antibody levels at diagnosis and viral loads inferred from PCR Ct values (a surrogate of infectivity). We also found evidence of ongoing transmission between vaccinated staff members, while no secondary transmission was documented in the Israeli study. The difference in the responsible variants (Alpha in the Israeli study and Delta in present study) might explain the different findings. Additionally, our study also showed no correlation between neutralizing antibody levels at diagnosis and the development of symptoms. Collectively, while neutralizing antibodies might be a surrogate of protection, especially against severe diseases as a whole [24], they might not be good indicators of infection progression and infectiousness for breakthrough Delta variant infection.

At the beginning of the outbreak, none of the HTD members of staff (including the PCR confirmed cases) tested positive for N-protein antibodies, which only develop in response to whole-virus based vaccine and natural infection. Additionally, between 12^th^ and 14^th^ May 2021, all members of HTD staff were subjected to a periodic testing for SARS-CoV-2 by PCR, but none was positive. These features suggest that the infected cases were captured at an early phase of the infection. Therefore, by carefully following up the patients during hospitalization, we have also provided new insights into the natural history of breakthrough Delta variant infections. We found viral loads of breakthrough Delta variant infection cases were 251 times higher than those of the infected cases detected during the early phase of the pandemic in 2020^7^, with high viral loads recorded around 2-3 days before and after the development of symptoms. Notably, a recent report from the US showed comparable viral loads between vaccinated and unvaccinated individuals infected with the Delta variant.^5^ Additionally, a study from China showed viral loads in people infected with the Delta variant were 1000 times higher than those in individuals infected with SARS-CoV-2 19A/19B strains detected in China in early 2020 [25]. Collectively, high viral loads might explain the current rapid expansion of the Delta variant, even in the countries with high vaccination coverage. Additionally, previous reports showed short duration of PCR positivity or culturable viruses (2–7 days after diagnosis) among breakthrough infections [26,27]. In contrast, we found prolonged PCR positivity was up to 33 days in our study participants.

Our study has several limitations. First, we only obtained SARS-CoV-2 genomes from 21/62 study participants, leaving the responsible variants in the remaining infected cases unknown. Second, we only focused our analysis on a hospital setting for a duration of two weeks. Therefore, our findings might not be generalizable for the general population, of which exposure to the virus might be different. Additionally, the short duration of the study coupled with the uncertain exposure to the virus prevented us from quantifying the risk of infection between vaccinated and unvaccinated individuals [28]. Third, we did not perform virus isolation to assess the duration of viral shedding, relying instead on PCR Ct values as a surrogate of infectivity. Fourth, information about other potential confounders (including BMI) was not available for inclusion in the case-control analysis. Last, none of the infected staff members was older than 60 years. Therefore, milder disease might be anticipated and breakthrough Delta variant infections in older people with comorbidities may be more severe.

In summary, we report SARS-CoV-2 Delta variant breakthrough infections among vaccinated health care workers with likely transmission between them. Most experienced mild symptoms and all recovered uneventfully. The infections were associated with high viral loads, prolonged PCR positivity, and low levels of neutralizing antibodies after vaccination and at diagnosis.

## Contributors

Study design: NVVC, NTD, LMH, NTT, GT, LVT

Study implementation and patient enrolment: VMQ, DBK, NTP, HPT, NTNM, NCH, LMH, LMT, LMY

Data collection: NTMT, TNHT, NHTT, NTP, DBK, VMQ

Laboratory testing: LAN, BTTT, VVP, TNPT, VTV, TTTT, NTT, HTB, HTKN, LTTU

Sequencing: NTTH, NTKT, LNTN, NTA, NTHN

Data analysis: VMQ, TTT, MC, LVT, NTA, NTTH, NMN, DTBT, DNHM

Laboratory supervision: DTBT, DNHM, NMN

Access to data and data verification: NVVC, DBK, VMQ, NTP, NTMT, NMN, MC, TTT, GT, LVT

Manuscript drafting, editing and writing: LVT

Manuscript editing: NVVC, TTT, MC, GT, VMQ

Funding acquisition: GT and LVT

All authors reviewed, approved, and agreed to submit the final version of the manuscript for publication.

## Funding

This study was funded by the Wellcome Trust of Great Britain (106680/B/14/Z, 204904/Z/16/Z and 222574/Z/21/Z) and NIH/NIAID (HHSN272201400007C and Subcontract No. S000596-JHU).

## Data sharing

SARS-CoV-2 sequences have been submitted to GISIAD (gisaid.org). De-identified study data are available for access by accredited researchers in accordance with data sharing policies of OUCRU.

## OUCRU COVID-19 Research Group

**Hospital for Tropical Diseases, Ho Chi Minh City, Vietnam**: Nguyen Van Vinh Chau, Nguyen Thanh Dung, Le Manh Hung, Huynh Thi Loan, Nguyen Thanh Truong, Nguyen Thanh Phong, Dinh Nguyen Huy Man, Nguyen Van Hao, Duong Bich Thuy, Nghiem My Ngoc, Nguyen Phu Huong Lan, Pham Thi Ngoc Thoa, Tran Nguyen Phuong Thao, Tran Thi Lan Phuong, Le Thi Tam Uyen, Tran Thi Thanh Tam, Bui Thi Ton That, Huynh Kim Nhung, Ngo Tan Tai, Tran Nguyen Hoang Tu, Vo Trong Vuong, Dinh Thi Bich Ty, Le Thi Dung, Thai Lam Uyen, Nguyen Thi My Tien, Ho Thi Thu Thao, Nguyen Ngoc Thao, Huynh Ngoc Thien Vuong, Huynh Trung Trieu Pham Ngoc Phuong Thao, Phan Minh Phuong

**Oxford University Clinical Research Unit, Ho Chi Minh City, Vietnam**: Dong Thi Hoai Tam, Evelyne Kestelyn, Donovan Joseph, Ronald Geskus, Guy Thwaites, Ho Quang Chanh, H. Rogier van Doorn, Ho Van Hien, Ho Thi Bich Hai, Huynh Le Anh Huy, Huynh Ngan Ha, Huynh Xuan Yen, Jennifer Van Nuil, Jeremy Day, Katrina Lawson, Lam Anh Nguyet, Lam Minh Yen, Le Dinh Van Khoa, Le Nguyen Truc Nhu, Le Thanh Hoang Nhat, Le Van Tan, Sonia Lewycka Odette, Louise Thwaites, Marc Choisy, Mary Chambers, Motiur Rahman, Ngo Thi Hoa, Nguyen Thanh Thuy Nhien, Nguyen Thi Han Ny, Nguyen Thi Kim Tuyen, Nguyen Thi Phuong Dung, Nguyen Thi Thu Hong, Nguyen Xuan Truong, Phan Nguyen Quoc Khanh, Phung Le Kim Yen, Phung Tran Huy Nhat, Sophie Yacoub, Thomas Kesteman, Nguyen Thuy Thuong Thuong, Tran Tan Thanh, Vu Thi Ty Hang

## Declaration of Competing Interest

All authors declare no competing interests.

## Appendix Supplementary materials

Image, application 1
