# Supplementary material for: An observational study of breakthrough SARS-CoV-2 Delta variant infections among vaccinated healthcare workers in Vietnam
Source: eClinicalMedicine. 2021 Sep 30;41:101143. doi: 10.1016/j.eclinm.2021.101143 (PMC8481205; doi:10.1016/j.eclinm.2021.101143)
Supplement: Supplementary file 1 [file mmc1.docx]

**SUPPLEMENTARY MATERIALS**

**Supplementary Table 1**: Demographics comparison between 30 matched uninfected controls and 10 infected staff members

|  | 10 cases | 30 matched uninfected controls* | P value |
| --- | --- | --- | --- |
| Age, y, median (IQR) | 45 (36-54) | 37 (34-46) | 0.145 |
| Male/female, n/n | 6/4 | 16/14 | 1.0 |
| Blood sampling after dose 2, d (median: IQR) | 17.5 (14-26) | 18 (16-21) | 0.74 |
| Blood sampling from dose 2 to diagnosis, d (median: IQR) | 51.5 (50-52) | 47 (43-49) | 0.001 |

**Notes to Supplementary Table 1:** *controls were those fully vaccinated and having blood collected at 14 after dose 2, of these 2 did not have blood collected at month three after dose 1 for neutralizing antibody measurement. Thus 28 were used for case-control analysis of at-diagnosis timepoint.

**Supplementary Table 2**: Demographics comparison between 30 controls who were tested positive for SARS-CoV-2 in Vietnam between March and April 2021and 62 cases

|  | 62 cases | 30 controls | P value |
| --- | --- | --- | --- |
| Male/female | 33/29 | 15/15 | 0.826 |
| Age, y, median (IQR) | 42 (32-50) | 31 (21-48) | 0.008 |
| Symptomatic | 49 (79)* | 17 (57)** | 0.026 |
| Comorbidity, n (%) | 17 (27) | 7 (23) | 0.656 |

**Notes to Supplementary Table 2**: *details can be found in Table 1, **symptomatic cases were those presenting with mild respiratory infection without oxygen

**Supplementary Table 3**: Numbers of PCR confirmed cases detected per department

| **Name of department*** | **Functions** | **Number of staff** | **Number of staff tested positive (%)** | **Numbers genomes obtained** |
| --- | --- | --- | --- | --- |
| Department A | Supportive service | 7 | 7 (100) | 6 |
| Department B | Supportive service | 56 | 16 (29) | 5 |
| Sub-department B1 | Supportive service | 8 | 7 (88) | 5 |
| Sub-department B2 | Supportive service | 7 | 4 (57) | 0 |
| Sub-department B3 | Supportive service | 8 | 3 (38) | 0 |
| Sub-department B4 | Supportive service | 9 | 2 (22) | 0 |
| Department C | Supportive service | 3 | 3 (100) | 3 |
| Department D | Supportive service | 60 | 12 (20) | 3 |
| Department E | Patient care | 75 | 7 (9) | 1 |
| Department F | Supportive service | 36 | 4 (11) | 0 |
| Department G | Patient care | 50 | 3 (6) | 0 |
| Department H | Supportive service | 20 | 3 (15) | 0 |
| Department I | Supportive service | 6 | 2 (33) | 1 |
| Department J | Patient care | 28 | 1 (4) | 1 |
| Department K | Patient care | 31 | 1 (3) | 0 |
| Department L | Patient care | 32 | 2 (6) | 0 |
| Department N | Patient care | 28 | 1 (4) | 0 |
| Department O | Patient care | 19 | 1 (5) | 1 |
| Department P | Patient care | 29 | 1 (3) | 0 |
| Department Q | Supportive service | 11 | 1 (9) | 0 |
| Department R | Supportive service | 15 | 1 (7) | 1 |
| Department S | Patient care | 17 | 1 (5.9) | 0 |
| Department T | Patient care | 18 | 1 (5.6) | 0 |
| Department U | Supportive service | 82 | 1 (1) | 0 |

**Supplementary Table 4:** Comparison between the 10 cases included for case-control analysis of neutralizing antibodies and 52 who was not included

|  | 10 case included for case-control analysis | 52 cases not included for case-control analysis | P value |
| --- | --- | --- | --- |
| Male/female | 6/4 | 27/30 |  |
| Age, y, median (IQR) | 45 (36-54) | 41 (32-49) | 0.276 |
| Symptomatic, n (%) | 8 (80) | 41 (79) | 0.237 |
| Comorbidity, n (%) | 4 (40) | 13 (25) | 0.440 |
| COVID-19 vaccination, n (%) | 10 (100) | 52 (100) |  |
| Two doses | 10 (100) | 50 (96) | 1.0 |
| One dose | 0 | 2 (4) |  |
| Inhibition at admisison, % (IQR) | 59.4 (32.5-73.2) | 72.7 (51.7-85)* | 0.110 |

**Note to Supplementary Table 4:** *n=49 who were fully vaccinated and had a blood sample collected at diagnosis.

**
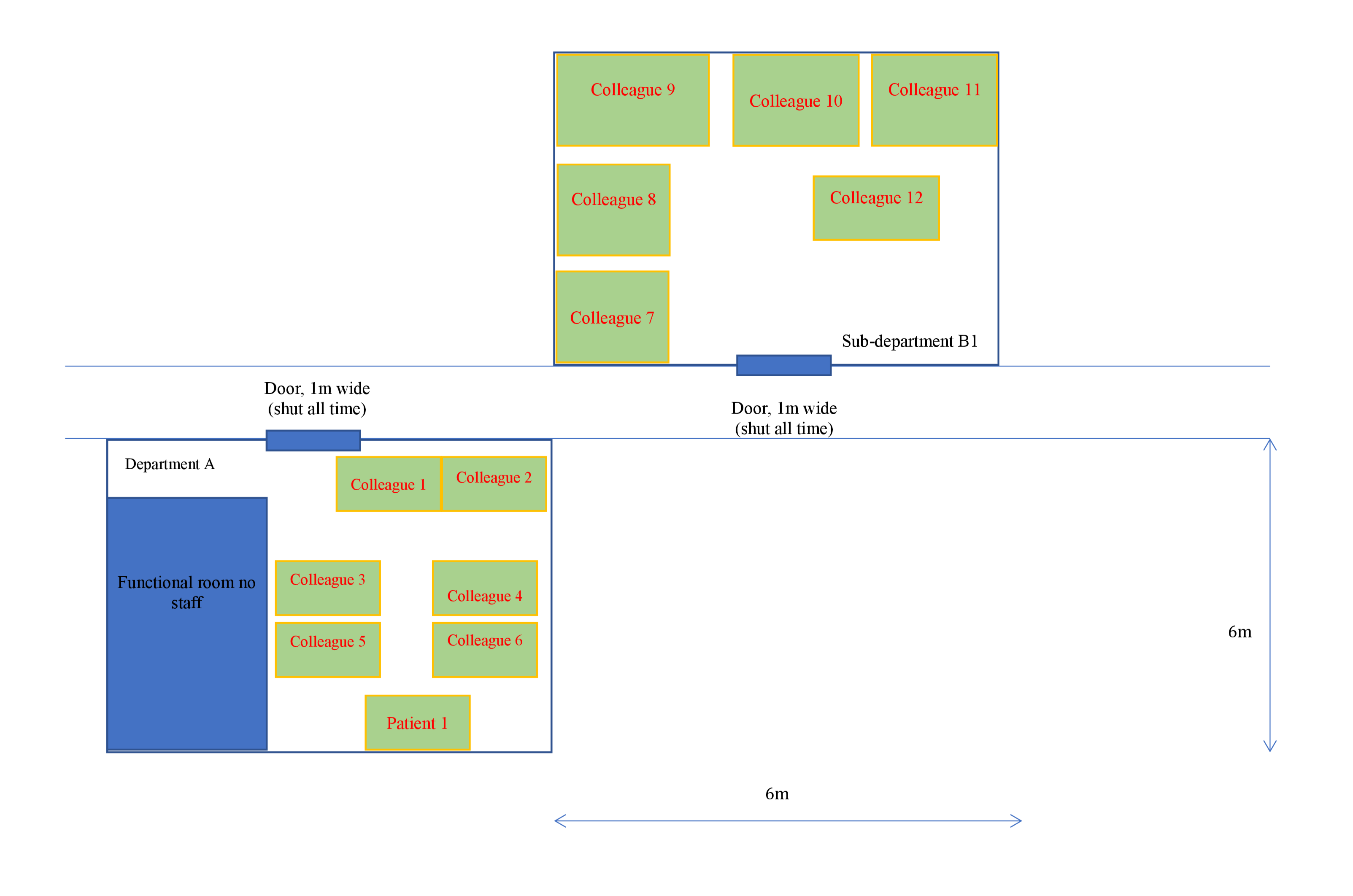
**

**Supplementary Figure 1**: Layout of office of patient 1 and a close office where 7/8 members were tested positive on 11^th^-12^th^ June 2021. Office names are linked with Supplementary Table 1. Offices are equipped with air conditioners without mechanical ventilation. During working hours, doors are kept closed to maintain air cooling.

**
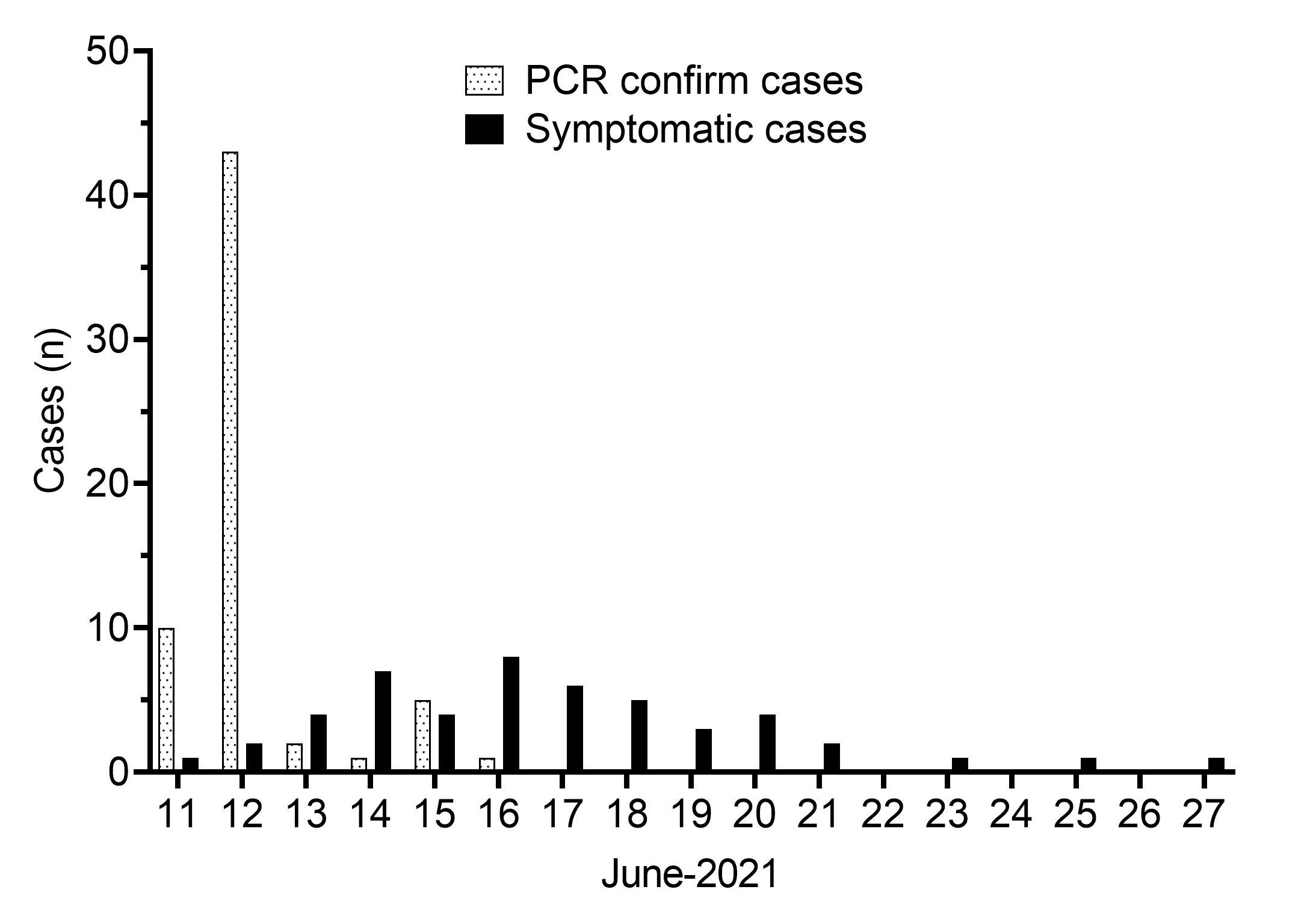
**

**Supplementary Figure 2:** Epidemic curve showing the distributions of the PCR confirmed and symptomatic cases. Symptomatic cases were plotted according to first date when they developed symptoms.


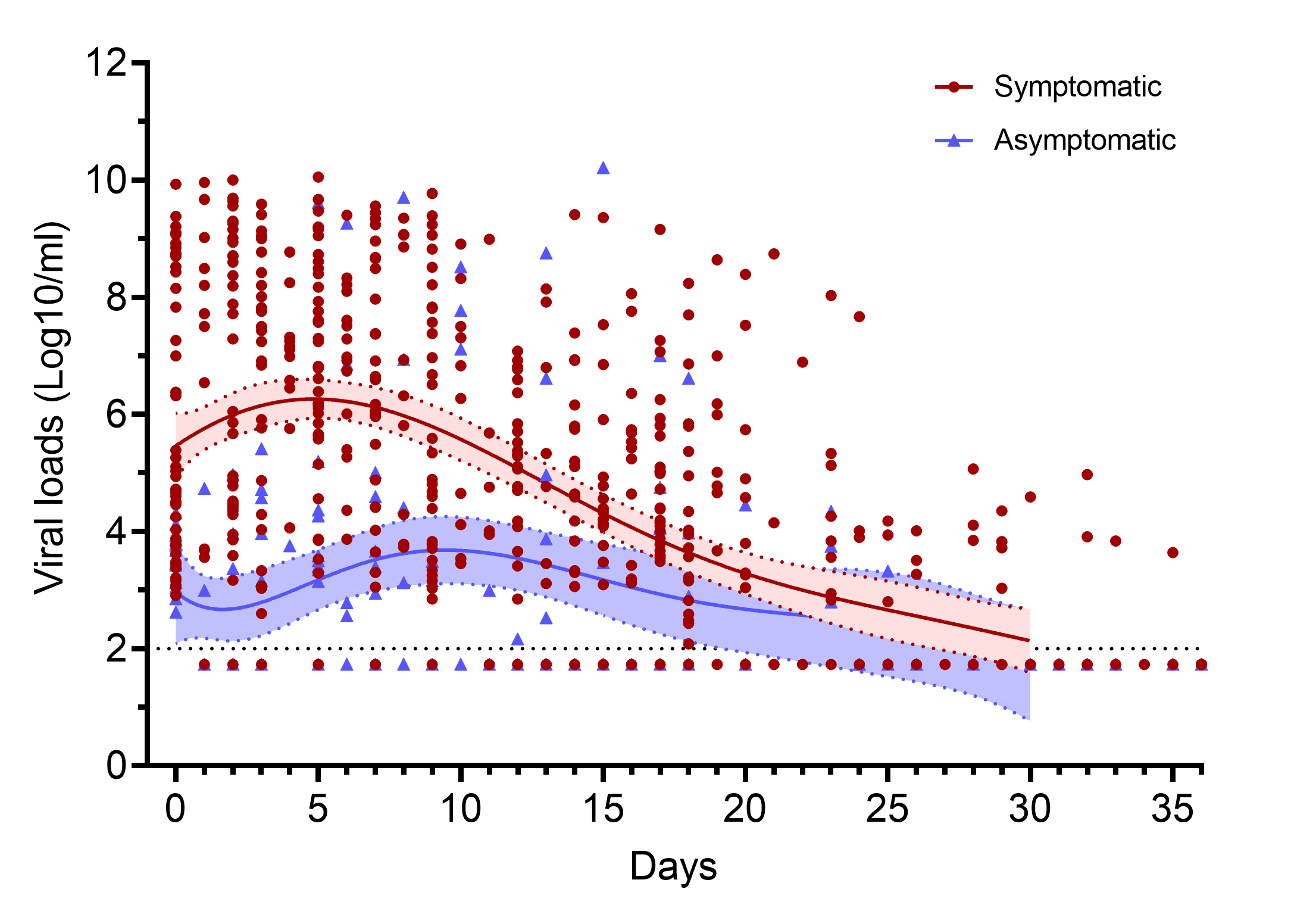


**Supplementary Figure 3**: Plot outlining kinetics of viral loads since PCR diagnosis during the course of hospitalization of the asymptomatic and symptomatic cases

**Notes to Supplementary Figure 3**: Lines indicate medians and shades indicate 95% confidence intervals.


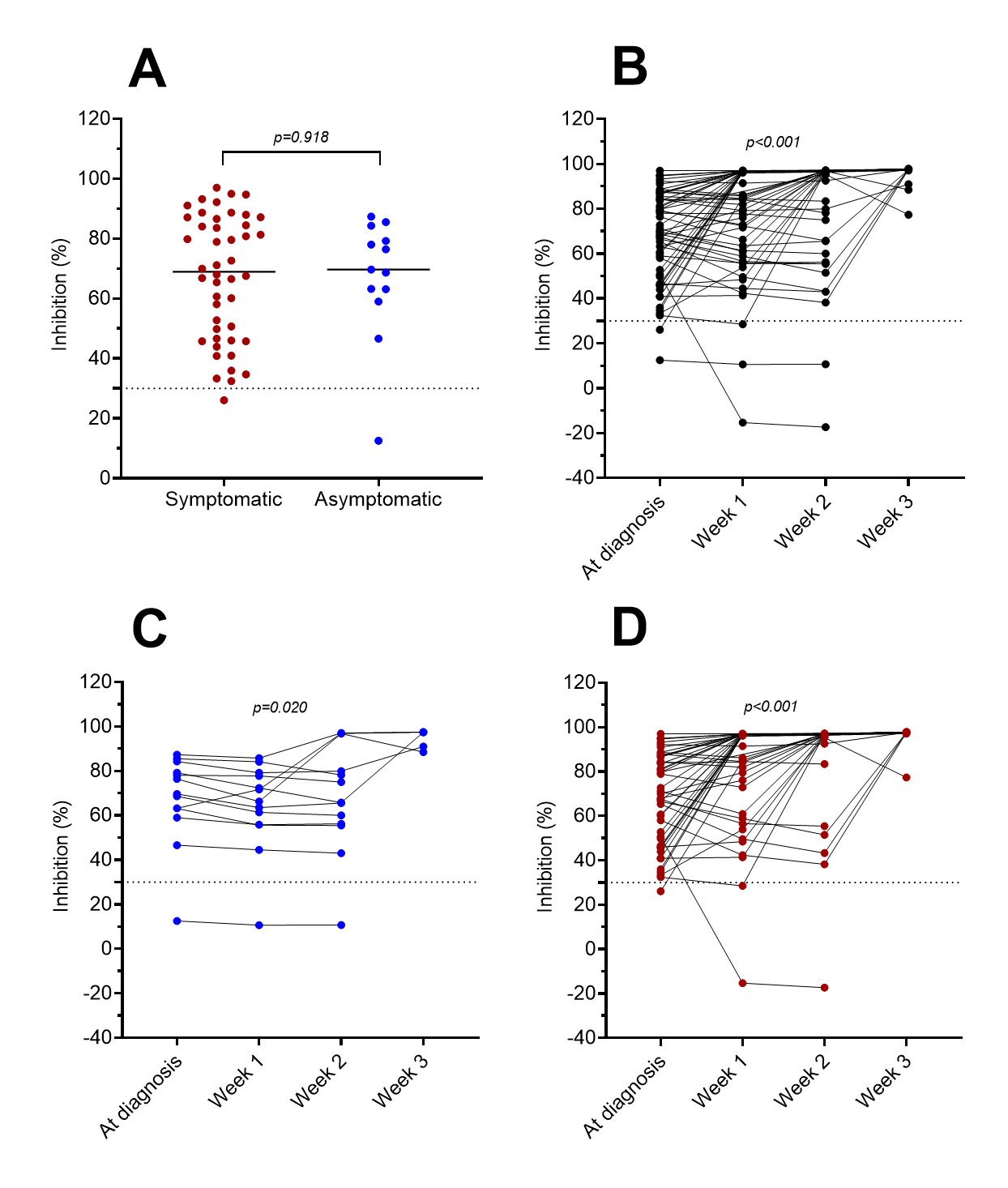


**Supplementary Figure 4**: Results of neutralizing antibody measurement, A) at diagnosis of symptomatic (including those developed symptoms after diagnosis) and asymptomatic cases, and kinetics of neutralizing antibodies at admission and at week 1, 2 and 3 after admission of B) the whole group, C) the asymptomatic group, D) the symptomatic group

**Supplementary Notes to Figure 4**: Dashed line indicates assay cut-off (30%). The asymptomatic case (panel C) who remained seronegative during infection did not respond to the vaccine (data not shown). Neutralizing antibody measurement were repeated twice for the symtomatic case who became seronegative at week 1 and week 2.


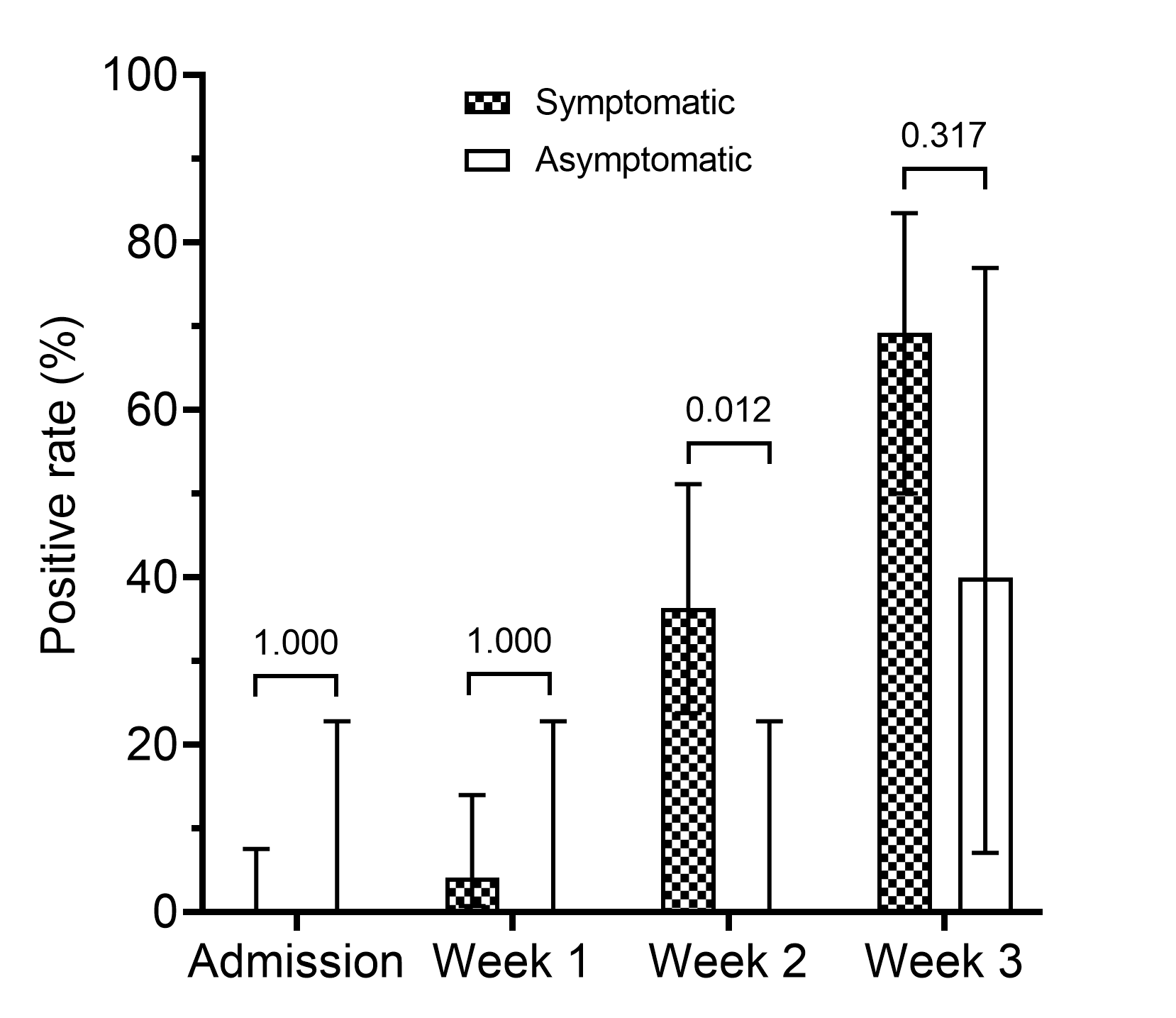


**Supplementary Figure 5**: Seroconversion rates against N protein at admission, and week 1, 2 and 3 after admission.

**Note to Supplementary Figure 5:** For the whole group, the seroconversion rates for antibodies against N protein increased from 0% at baseline to 3.3% (2/61) at week 1, 28.1% (16/57) at week 2 and 65% (20/31) at week 3.
